# Coexistence of EZH2, NOTCH1, IL7R, and PHF6 Mutations in Adult T-cell Acute Lymphoblastic Leukemia

**DOI:** 10.4274/tjh.2017.0194

**Published:** 2017-12-01

**Authors:** Xilian Zhou, Yan Gu, Qi Han, Mario Soliman, Chunhua Song, Zheng Ge

**Affiliations:** 1 Zhongda Hospital, Medical School of Southeast University Department of Hematology Nanjing, China; 2 Pennsylvania State University, Department of Pediatrics, Pennsylvania, USA

**Keywords:** EZH2, Adult, T-cell, Acute lymphoblastic leukemia

## To The Editor,

**Figure 1:**
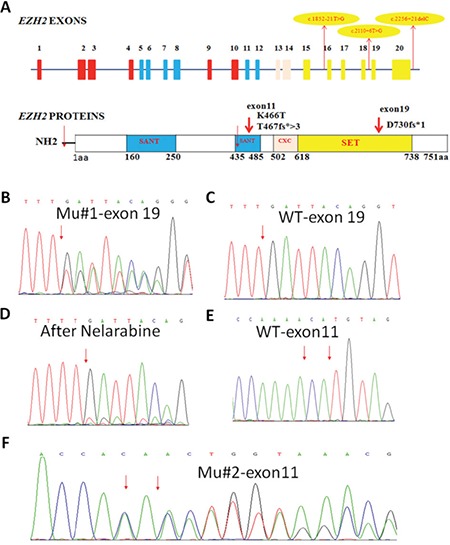
Location and sequencing data of the EZH2 mutations. A) Mutation 1 (Mu#1:D730fs*1), located in exon 19, is a frame shift-creating insertion; on the protein level, it leads to a truncated protein with a length of 731 amino acids, which is located in the conserved catalytic SET domain(amino acids 618-731). This domain is critical for the methyltransferase activity of EZH2. The other two mutations (Mu#2:K466T; Mu#3:T467fs*>3)located within exon 11 are anon-synonymous single-nucleotide substitution and a frame shift-creating deletion, respectively; on the protein level, they result in the substitution of EZH2 lysine466 to tyrosine and a truncation of the EZH2 protein, respectively. Mu#2 and Mu#3 are novel EZH2 mutations; both of them are located in the SANT domain of the EZH2 protein (amino acids 435-485), which is known to be incharge of the DNA binding. Mu#1 was detected in patient 1 and the other two in patient 2. Blue, pink, and yellow bars correspond to exons encoding the SANT domains, the cysteine-rich CXC domain, and the SET domain, respectively. The red arrows show EZH2 mutations. B-F)The direct sequencing data of EZH2 mutations (B, D, F) and wild-type (C, E). B: c.2187_2188insT p.D730fs*1; C: EZH2 exon 19 wild-type; D: Mu#1 after nelarabine treatment; E: EZH2 exon 11 wild-type; F: c.1397A>C; 1399delA p.K466T; T467fs*>3.

Enhancer of zestehomolog 2 (EZH2) mutations are reported in solid tumors [1,2,3] as well as leukemia, and they are most commonly detected in early T-cell precursor acute lymphoblastic leukemia (ETP-ALL) [4,5,6,7,8], which is an extraordinarily aggressive malignancy of enigmatic genetic basis [9]. We screened EZH2 mutations in 146 Chinese adult ALL patients, among which 24.7% (36/146) cases were T-cell acute lymphoblastic leukemia (T-ALL) and 12.9% (4/31) T-ALL cases were identified as ETP-ALL. We found three EZH2 mutations in two patients with T-ALL. One patient had Mu#1:D730fs*1, a truncation mutation that was previously reported in acute myeloid leukemia, and the another patient had two new EZH2 mutations, Mu#2:K466T and Mu#3:T467fs*>3 (Figure 1). We also screened the mutations in other genes (Table 1). Strikingly, the EZH2 mutations coexisted with mutations of NOTCH1, IL7R, and PHF6 in the two patients and they responded poorly to chemotherapy and experienced difficult clinical histories and inferior outcomes (Table 1). Patient 1 was diagnosed with T-ALL with myeloid expression based on his bone marrow (BM) smear and immunophenotypes (Table 1). With the first inductive therapy (Table 1), the patient achieved complete remission (CR) with 0.1% blasts in the peripheral blood (PB) and 0.8% in BM. One year later, the patient relapsed with 90.4% lymphoblasts in the BM and 1.0% in the PB, and CR was achieved after the first chemotherapy. During the following treatment, he underwent an intramedullary and an extramedullary relapse infiltrating his left tonsil and then endured three more relapses. On the fifth relapse, the BM blast rate was 50.4%. Although the patient was treated with nelarabine, no CR was achieved in the subsequent treatments. Even though the BM blast rate was 5.2%, the patient died of infection during the BM suppression period after he received the last chemotherapy. We examined the EZH2 mutational status in the BM samples of the 1st relapse, 5th relapse, and 6 weeks after his 5th relapse; the EZH2 and NOTCH1 mutation status remained the same as in the first diagnosis even after the nelarabine treatment (Figure 1D). Patient 2 presented with 80.0% lymphoblasts in the PB and 78.0% blasts in the BM (Table 1). Two somatic mutations, K466T and T467fs*>3 in EZH2 exon 11, were detected in her BM sample (Figure 1). No CR was achieved with the first induction therapy. Finally, the patient was administered methotrexate and cytarabine and endured a long period of BM suppression. Unfortunately, the patient was lost to follow-up. Our data indicated the oncogenic and poor prognostic effect of EZH2 mutations on T-ALL. The coexistence of EZH2 mutations with mutations in the NOTCH1, PHF6, and IL7R genes suggested a new mechanism underlying the tumorigenesis of EZH2 mutations in T-ALL. T-ALL and particularly ETP-ALL still have largely negative outcomes. In the past years, the effect of the use of nelarabine for relapsed and refractory T-ALL seemed to be negligible [10]. In our cohort, the first patient’s relapse, even after nelarabine treatment, revealed the insensitivity of patients with multiple mutations to such treatment. Moreover, our case report suggested that the gene mutations may be the cause of the failure of the drug treatment and emphasized the importance of developing more effective therapies as well as more active and tailored treatments for aggressive T-ALL.

## Figures and Tables

**Table 1:**
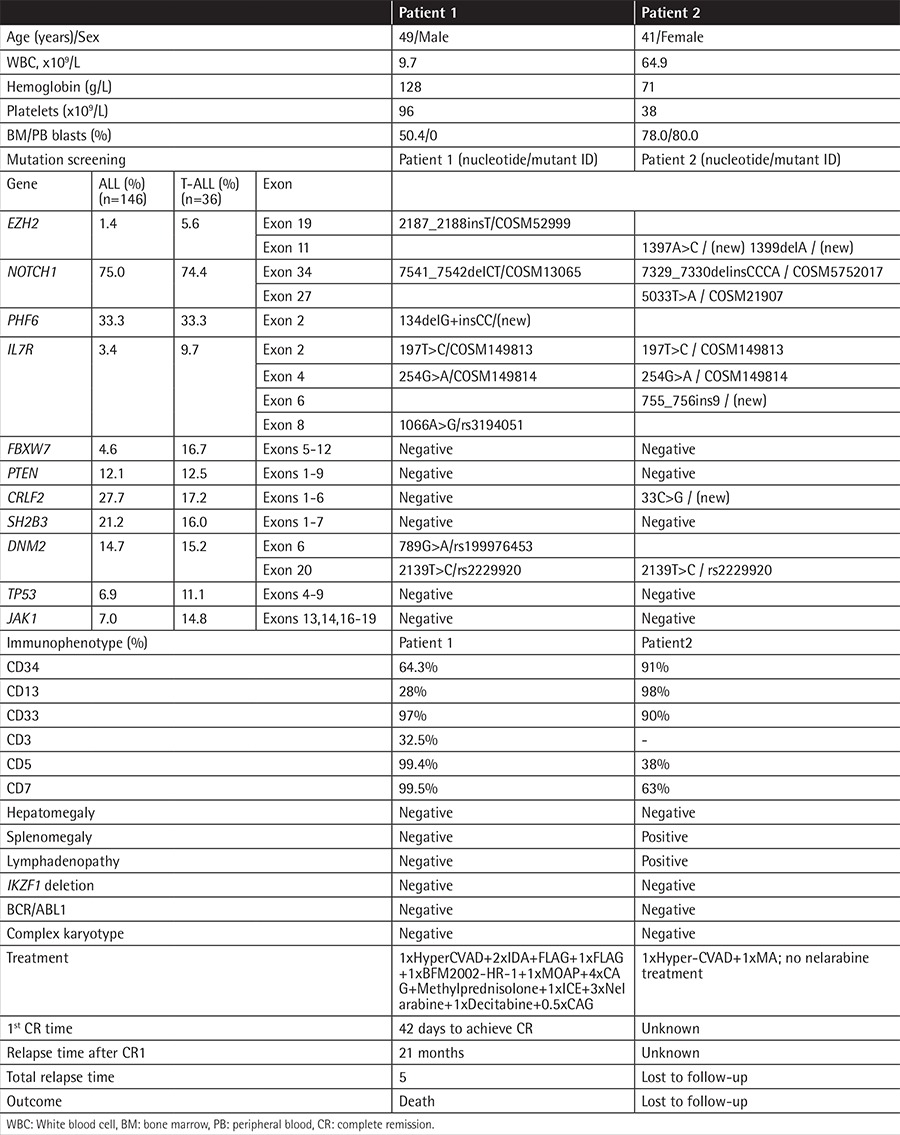
Clinical features of patients with EZH2 mutations.
